# Rust Secreted Protein Ps87 Is Conserved in Diverse Fungal Pathogens and Contains a RXLR-like Motif Sufficient for Translocation into Plant Cells

**DOI:** 10.1371/journal.pone.0027217

**Published:** 2011-11-04

**Authors:** Biao Gu, Shiv D. Kale, Qinhu Wang, Dinghe Wang, Qiaona Pan, Hua Cao, Yuling Meng, Zhensheng Kang, Brett M. Tyler, Weixing Shan

**Affiliations:** 1 College of Plant Protection, Northwest A&F University, Yangling, Shaanxi, China; 2 Viginia Bioinformatics Institute, Blacksburg, Virginia, United States of America; 3 College of Life Science, Northwest A&F University, Yangling, Shaanxi, China; 4 State Key Laboratory of Crop Stress Biology for Arid Areas, Northwest A&F University, Yangling, Shaanxi, China; University of Minnesota, United States of America

## Abstract

**Background:**

Effector proteins of biotrophic plant pathogenic fungi and oomycetes are delivered into host cells and play important roles in both disease development and disease resistance response. How obligate fungal pathogen effectors enter host cells is poorly understood. The *Ps87* gene of *Puccinia striiformis* encodes a protein that is conserved in diverse fungal pathogens. Ps87 homologs from a clade containing rust fungi are predicted to be secreted. The aim of this study is to test whether Ps87 may act as an effector during *Puccinia striiformis* infection.

**Methodology/Principal Findings:**

Yeast signal sequence trap assay showed that the rust protein Ps87 could be secreted from yeast cells, but a homolog from *Magnaporthe oryzae* that was not predicted to be secreted, could not. Cell re-entry and protein uptake assays showed that a region of Ps87 containing a conserved RXLR-like motif [K/R]RLTG was confirmed to be capable of delivering oomycete effector Avr1b into soybean leaf cells and carrying GFP into soybean root cells. Mutations in the Ps87 motif (KRLTG) abolished the protein translocation ability.

**Conclusions/Significance:**

The results suggest that Ps87 and its secreted homologs could utilize similar protein translocation machinery as those of oomycete and other fungal pathogens. Ps87 did not show direct suppression activity on plant defense responses. These results suggest Ps87 may represent an “emerging effector” that has recently acquired the ability to enter plant cells but has not yet acquired the ability to alter host physiology.

## Introduction

Biotrophic plant pathogens such as rust and powdery mildew fungi form special feeding structures called haustoria, by which they intimately contact their hosts and secrete large numbers of effector proteins capable of entering into host cells. Secreted effector proteins can transfer across host plasma membranes to reach their destinations where they manipulate plant cellular components, including the defense system, to facilitate pathogen colonization and proliferation [1]. Most effectors of fungal and oomycete plant pathogens identified so far are small secreted proteins with high sequence divergence that are expressed prior to or during infection [2], [3], [4], [5], [6].

Large-scale genome sequencing has led to the prediction of large numbers of proteins potentially secreted by pathogens as effectors. However, predicting the functions of such genes and whether the encoded proteins might enter host cells is still very difficult. A few effectors have been well characterized, especially those effectors called avirulence proteins that interact with plant resistance (R) gene products to trigger gene-for-gene resistance (also called effector-triggered immunity; ETI). For instance, the *Avr-Pita* effector from rice blast fungus *Magnaporthe oryzae* encodes a putative zinc-dependent metalloprotease [2], [7] and the AvrP123 protein of the flax rust fungus *Melampsora lini* is predicted to be a Kazal-like protease inhibitor based on computational analysis [8]. The application of high throughput bioassays, notably the *Agrobacterium tumefaciens*-mediated transient expression and virus-induced or RNAi-dependent gene silencing, have provided useful tools for functional analysis of the predicted secreted proteins [9], [10], [11], [12]. Many putative secreted proteins of biotrophic fungi and oomycetes were found to have activities of either cell death induction or cell death suppression important for their contributions to pathogenesis [5], [11], [13], [14].

Many intracellular effectors were inferred to be able to enter the cytoplasm of plant cells after secretion because of the intracellular locations of their corresponding resistant gene products [15], [16], [17], [18], [19]. Bacterial effectors may be injected into plant cells via several secretion systems [20]. Nematode effectors enter host cells via the stylet [21]. A mechanism for translocation of oomycete and fungal effectors has recently been uncovered. Most identified oomycete effectors carry a conserved N-terminal RXLR motif, which is necessary and sufficient to mediate entry of effectors inside host cells in the absence of pathogens [22], [23] via binding to the lipid phosphatidylinositol-3-phosphate (PI-3-P). Meanwhile, effectors of both oomycete and fungi, including a rust pathogen and an ectomycorrhizal fungus, were demonstrated to contain functional RXLR variants which could mediate their transduction into plant cells in the absence of the pathogen or the symbiont [24], [25], [26], [27], in some cases via PI-3-P-binding [24], [27]. Thus RXLR-like domains may be widely responsible for translocation of fungal effectors.

We show that a predicted effector, Ps87 of *Puccinia striiformis* f. sp *tritici* (GenBank accession number: ES322018.1), can be secreted and can enter plant cells via a conserved RXLR-like motif KRLTG. Homologs of Ps87 in ascomycete fungi are however not predicted to be secreted, which we confirm for the *Magnaporthe oryzae* homolog. Our results suggest that Ps87 proteins in rust fungi may represent functional diversification of an intracellular protein into a secreted effector.

## Materials and Methods

### Microbial strains, plant materials and growth conditions

For preparation of the plasmids, *Escherichia coli* DH10B competent cells were used; for transient expression of constructs in *Nicotiana benthamiana*, *Agrobacterium tumefaciens* strain GV3101 was used with appropriate antibiotics. All bacterial DNA transformations were conducted by electroporation using standard protocols. The invertase secretion-deficient yeast strain YTK12 [11] was used for signal peptide validation. *Phytophthora parasitica* strain Pp106 used for inoculation was routinely cultured in carrot agar medium [28]. *A. tumefaciens* was cultured at 28°C, *E. coli* at 37°C, *Saccharomyces cerevisiae* at 30°C, and *P. parasitica* at 25°C, all in growth chambers in the dark.

Susceptible soybean cultivar Williams and resistant soybean cultivar L77-1863 with the *Rps1b* gene were used for transient transformation by particle bombardment; Williams was used for the root uptake assay [23]. *Nicotiana benthamiana* plants were used for transient transformation by *Agrobacterium*-mediated transformation of leaf tissues. Soybean and *N. benthamiana* were grown at 28°C during the light period and 25°C during the dark period. Light conditions averaged 4000 Lux, at 16 hr day and at 8 hr night.

### Sequence Analysis

Similarity searches were performed locally using standard bioinformatics programs such as NCBI blastp. Sequences of Ps87 homologs in diverse fungal pathogens were analyzed for predicted signal peptides using three different *in silico* programs, SignalP3.0, iPSORT and PSORTII. When using the SignalP3.0 program, HMM prediction scores higher than the value of Hesp-767 [8] were deemed as potential signal peptides. Phylogenetic relationships were inferred using the neighbor-joining method.

### Plasmids and strain construction

The primers list and cloning strategies for all plasmids involved are summarized in the supporting information Table S1.

### Yeast signal peptide screen

Functional validation of the predicted signal peptide of Ps87 was conducted with a yeast secretion system [29]. The yeast signal trap vector pSUC2T7M13ORI (pSUC2), which carries a truncated invertase, SUC2, lacking both its initiation methionine and signal peptide, was used. Yeast cells were transformed with 0.5 µg of the individual pSUC2-derived plasmids using the lithium acetate method. DNA encoding the predicted signal peptide plus the two following amino acids of Ps87 or Avr1b, or the first 25 amino acids of the *M. oryzae* Ps87 homolog (Mg87) were cloned as an EcoRI–XhoI fragments into pSUC2 [11], [26], then transformed into the yeast strain YTK12. All transformants were confirmed by PCR with vector-specific primers. Transformants were grown on yeast minimal medium with sucrose in place of glucose (CMD-W medium: 0.67% yeast N base without amino acids, 0.075% tryptophan dropout supplement, 2% sucrose, 0.1% glucose, and 2% agar). To assay for invertase secretion, colonies were replica plated onto YPRAA plates containing raffinose and lacking glucose (1% yeast extract, 2% peptone, 2% raffinose, and 2 µg/mL antimycin A). The YTK12 transformed with the pSUC2 vector encoding the truncated invertase and untransformed YTK12 strain were used as negative controls.

### Particle bombardment assays for avirulence and virulence phenotypes

Particle bombardment assays were performed using a double-barreled extension of the Bio-Rad He/1000 particle delivery system [23], [24], [30] to determine if the N-terminal motifs of putative effectors could direct *Phytophthora sojae* Avr1b protein to enter the host cells by utilizing the host transduction machinery. Empty vector mixed with a plasmid encoding beta-glucuronidase (GUS) or a mixture of plasmids encoding the Avr1b fusion protein and GUS were delivered into host cell side by side via the double barrel gene gun. The fusion proteins consisted of a functional secretory signal peptide of Avr1b and a putative Ps87 protein translocation domain (wild-type or mutant) followed by the C-terminus of Avr1b. The signal peptide directs secretion of the fusion protein out of the cell. The putative protein translocation domain was tested to see if it is able to bring the protein back into the cell. Re-entry of the protein into the plant cells was measured by a reduction of GUS+ spots resulting from cell death triggered by the C-terminus of Avr1b in soybean leaves containing *Rps1b*. Leaves lacking *Rps1b* were used as a control. For each paired shot (GUS+Ps87(N)-Avr1b DNA versus GUS+control DNA), the logarithm of the ratio of the spot numbers with the fusion protein compared to that of the control was calculated, and then the log ratios obtained from the *Rps1b* and non-*Rps1b* leaves were compared by the Wilcoxon rank sum test.

### Expression and purification of His-tagged GFP fusion proteins

The BL21(DE3) *Escherichia coli* cells containing plasmids encoding each fusion proteins were grown in 200 mL of Luria-Bertani (LB) media containing 100 µg/mL ampicillin in a one-liter baffled flask shaken at 220 rpm at 37°C to an OD_600_ of 0.6. Then, for the following fusion proteins, expression was induced with 2.5 mM IPTG and cell growth was continued under the same conditions for 4 hr at 20°C: control GFP, Avr1b(N)-GFP and Avr1b(N)-GFP mutants. Expression of the following fusion proteins was induced with 1 mM IPTG and then cell growth was continued at 220 rpm at 20°C for 6 hr: Ps87(N)-GFP and Ps87(N)M8-GFP. Cells were harvested by centrifugation at 4°C at 6,000 g for 10 min and stored at −80°C. Resuspended cells were lysed by sonication in chilled lysis buffer (50 mM NaH_2_PO_4_ (pH 7.2), 300 mM NaCl, 10 mM imidazole, 1 mg/mL lysozyme; 4 mL/g wet cell weight). Lysed cells were centrifuged at 10,000 g for 30 min at 4°C and then fusion proteins were collected from the supernatant using a column containing Ni-NTA Resin (GenScript). The column was then washed with five volumes of washing buffer (50 mM NaH_2_PO_4_, 300 mM NaCl, 20 mM imidazole, pH 7.2) and eluted in 2 mL fractions with elution buffer (50 mM NaH_2_PO_4_, 300 mM NaCl, 300 mM imidazole pH 7.2). Fractions were pooled then concentrated to ∼100 µL and equilibrated to 25 mM MES (2-(N-morpholino)ethanesulfonic acid) at pH 5.8 (for the soybean root uptake assay) using a concentrator device (Sartorius Ultracentrifuge-6 with 10 kDa membrane). Protein concentrations were measured by absorbance at 280 nm using a Nanodrop spectrophotometer (ND-1000). The purity was assessed by gel electrophoresis.

### Soybean root protein uptake and confocal laser scanning microscopy

Soybean seeds of variety Williams were germinated in vermiculite for four to five days. Roots were washed with water thoroughly to remove any debris. Approximately 1 cm root tips were cut and placed into the protein solution (50–100 µL–25 mM MES pH 5.8, 1 mg/mL protein) and incubated for 12–15 hr at 28°C. Then the root tips were rinsed with water and washed in 75 mL of water for 2 hr on an orbital shaker at 120 rpm. Microscopy analysis was performed using a Zeiss LSM 510 laser-scanning microscope. For the root uptake assay, excitation of GFP was done at 488 nm with the argon laser tube current set to 4.5 Amp. Emission was captured using a 505–530 nm broad pass filter. The detector gain was between 540 and 700 with an amplifier offset of −0.2–0. Calibrations of gain settings were performed with multiple control roots with a range of background fluorescence to avoid capturing background fluorescence.

### Transient Agro-Infiltration assay

For *in planta* expression, *Agrobacterium tumefaciens* strain GV3101 with respective constructs were grown in LB media supplemented with 50 µg/mL of kanamycin and 20 µg/mL of rifampicin to late-log phase. The cells were collected by centrifugation (4000 rpm, 10 min, 10°C), and resuspended in an infiltration media (10 mM MgCl_2_, 10 mM MES, pH 5.6, and 200 mM acetosyringone), and then incubated at room temperature for 1–3 hr before infiltration. *A. tumefaciens* solutions were infiltrated at an OD_600_ of 0.3–0.6. Agroinfiltration experiments were performed on leaves of six- to eight-week-old *Nicotiana benthamiana* plants. Plants were grown and maintained throughout the experiments in a cultivation room with an ambient temperature of 22–25°C and high light intensity. Photographs were taken five days after infiltration. Defense-related genes transcriptional analysis was performed after agroinfiltration of AgmPs87 on *N. benthamiana* leaves. Four leaves of each treatment were collected after infiltration through a time course from one to three days. Total RNA was extracted from the pooled *N. benthamiana* leaf samples using the TRIZOL solution (Invitrogen). First-strand cDNA was synthesized using 2 µg of total RNA, oligo (dT) primer (Takara), and M-MLV reverse transcriptase (Takara) according to the manufacturer's instructions. cDNA was ten fold diluted and 1 µL was used per well in 20 µL reactions. To perform inoculation with pathogen mycelia, agar discs (1 cm in diameter) from the colony edges of one-week-old *P. parasitica* cultures were transferred onto infiltration site of *N. benthamiana* leaves, the susceptibility of the infiltrated leaves to pathogen infection was evaluated three days post-inoculation.

## Results

### The predicted secreted protein Ps87 of *Puccinia striiformis* is conserved in diverse fungal plant pathogens

**Figure 1:**
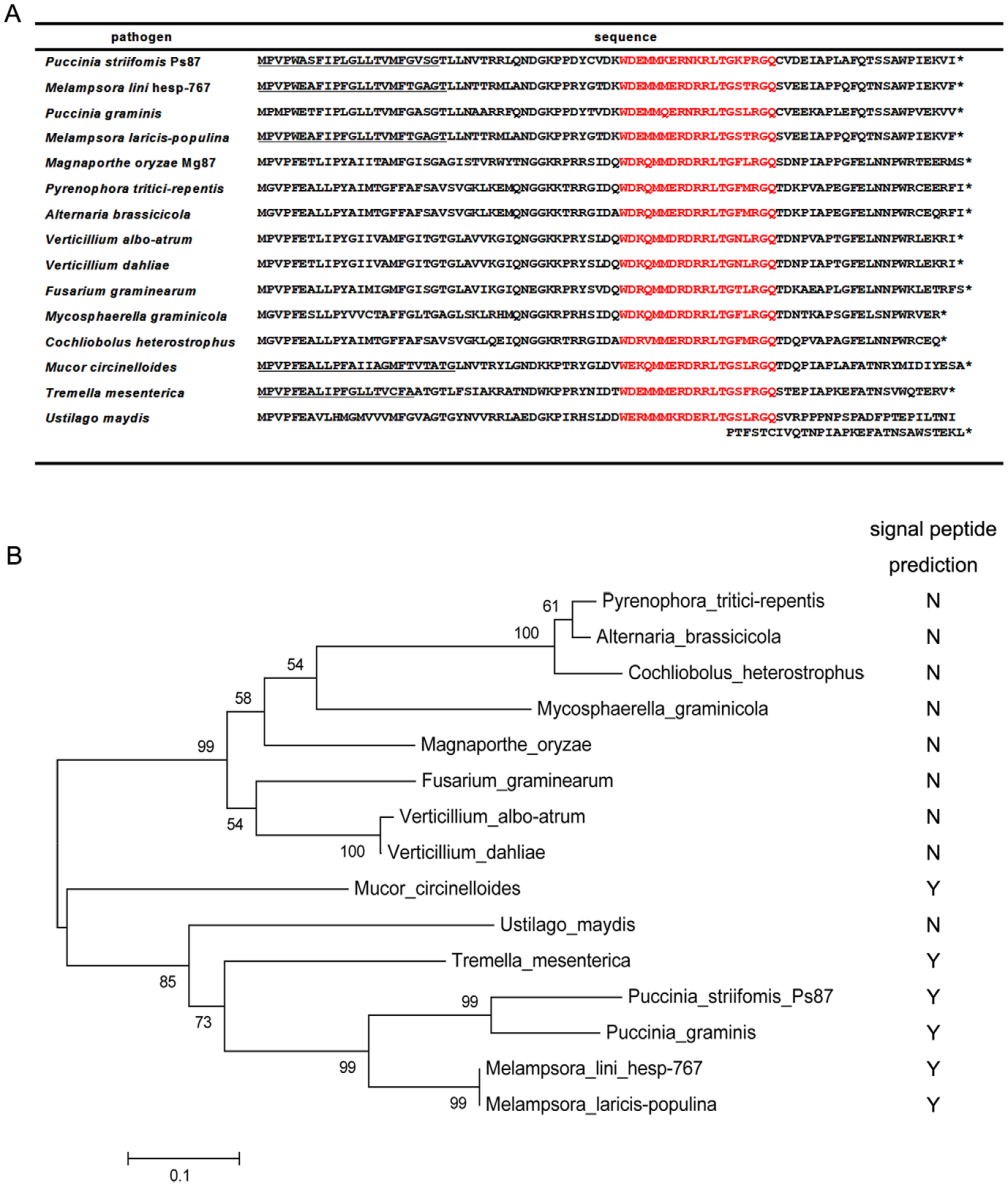
Sequence alignment and phylogenetic analysis of Ps87 and homologs. (A) Sequence alignment of Ps87 and homologs from other fungi. All the Ps87 homologs were collected through NCBI blast searches. Potential signal peptides (underlined) were predicted by signalP3.0. Sequence information for alignment can be found in the GenBank data library and JGI data library under accession numbers. *Fusarium graminearum*, XP_388656.1; *Magnaporthe oryzae* XP_362169.1; *Melampsora lini hesp-767*, ABB96277.1; *Melampsora laricis-populina*, EGG10381.1; *Mycosphaerella graminicola*, EGP88947.1; *Puccinia graminis* f. sp *tritici*, XP_003322973.1; *Pyrenophora tritici-repentis*, XP_001932799.1; *Verticillium albo-atrum*, XP_003000816.1; *Verticillium dahliae*, EGY16300.1; *Ustilago maydis*, XP_759190.1; *Alternaria brassicicola* jgi|5010; *Cochliobolus heterostrophus*, jgi|C88918; *Mucor circinelloides*, jgi|34515; *Tremella mesenterica*, jgi|67185. (B) Phylogenetic analysis of Ps87 and its homologs in diverse plant pathogens. Homologs with signal peptide HMM prediction scores higher than the value of hesp-767 are labeled as Y, else they are marked N. The phylogenetic relationship was inferred using the neighbor-joining method.

*Ps87* was an EST from the germinated urediniospore cDNA library of *P. striiformis* f. sp *tritici* [31]. Its corresponding transcript was up-regulated during urediniospore germination [31], *Ps87* was identified to encode a secreted protein with high similarity to the predicted haustorially expressed secreted protein 767 (hesp767) of *Melampsora lini* [8] (Figure 1A). Database searches led to the identification of Ps87 homologs in diverse fungal plant pathogens, such as *Puccinia graminis* f. sp *tritici*, *Melampsora lini*, *Melampsora laricis-populina*, *Magnaporthe oryzae*, *Alternaria brassicicola* and *Pyrenophora tritici-repentis* (Figure 1A). Phylogenetic analysis categorized Ps87 and homologs into two distinct subgroups (Figure 1B). An alignment of these proteins revealed an extremely conserved region of 19 amino acid residues near the middle of the predicted proteins (Figure 1A). The conservation of this protein in biotrophic and necrotropic plant pathogens suggested an important role in fungal biology and/or pathogenesis.

**Figure 2:**
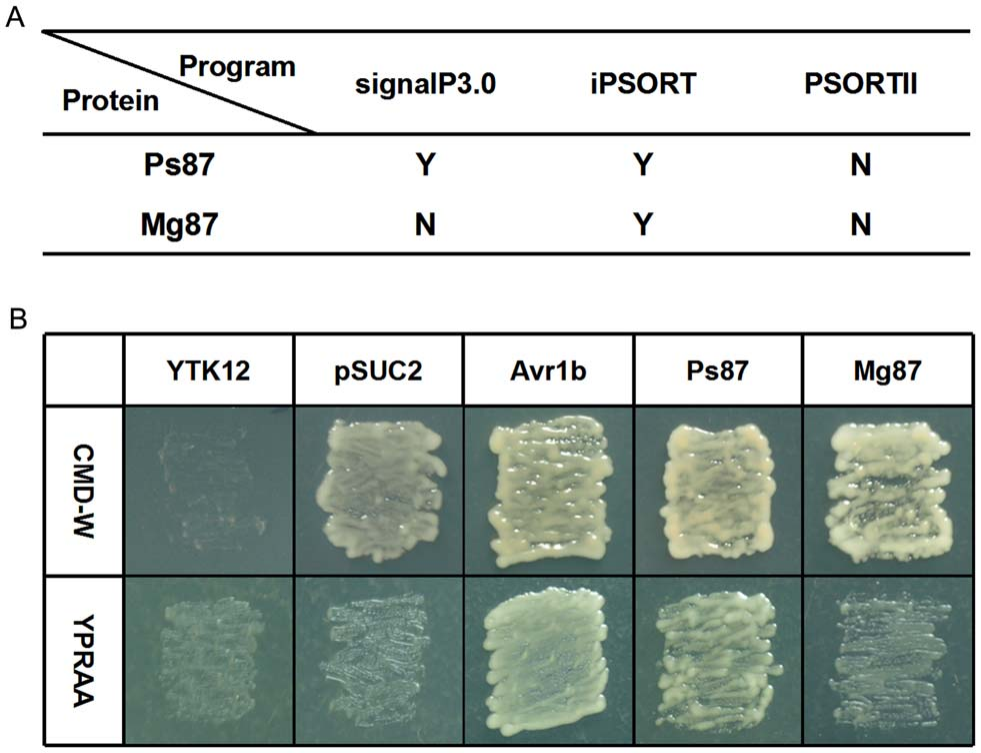
Prediction and functional validation of the signal peptide of *Puccinia striiformis* Ps87 protein. (A) Prediction of a secretory signal peptide in Ps87 and Mg87 by algorithms, signalP3.0, iPSORT and PSORTII. Positive predictions are labeled Y and negative ones, N. (B) Functional validation of the signal peptides of Ps87 and Mg87 using the yeast invertase secretion assay. The Ps87 or Avr1b signal peptides, or the first 25 amino acids of Mg87 were fused in frame to the invertase sequence in the pSUC2 vector and were transformed into yeast YTK12 strain. Controls include the untransformed YTK12 strain that and YTK12 carrying the pSUC2 vector. Strains are unable to secrete invertase can grow on CMD-W medium but not on YPRAA medium.

The hesp767 protein of flax rust fungus was previously annotated as a predicted secreted protein [8]. We analyzed homologs of Ps87 from a wide diversity of fungi using the prediction algorithm, SignalP3.0. The results revealed that only the predicted proteins that belong to rust fungi, including Ps87, and to *Mucor circinelloides* showed a positive prediction by SignalP3.0; all other proteins were predicted to be non-secreted by SignalP3.0 (Figure 1). Ps87 consisted of 85 amino acids and a signal peptide sequence was detected via SignalP3.0 and iPSORT signal peptide–predicting programs, but the signal peptide identification program PSORTII showed negative result (Figure 2A). Ps87 homolog of *M. oryzae* was recognized as non-secreted protein through both SignalP3.0 and PSORTII program but not iPSORT program.

### Functional validation of the signal peptide of *P. striiformis* Ps87

To functionally validate the SignalP3.0 predictions, the signal peptide of Ps87 was tested using a genetic assay based on the requirement of yeast cells for invertase secretion to grow on raffinose medium [11], [26], [29]. As a control we tested the N-terminus of the *Magnaporthe oryzae* homolog Mg87, which was predicted not to be secreted. The secretory leader of Avr1b was used as a positive control. The predicted signal peptide and the subsequent two amino acids of Ps87 and Avr1b were fused in frame to the mature sequence of yeast invertase in the vector pSUC2, respectively. The first 25 amino acids of Ps87 homolog Mg87 from *M. oryzae* were also cloned into pSUC2 and examined. Both *Ps87* and *Avr1b* fused constructs enabled the invertase mutated yeast strain to grow on CMD-W medium (yeast growth without invertase secretion) and YPRAA medium (growth only when invertase is secreted). By contrast, when the Mg87 N-terminus was fused to truncated invertase, the transformed yeast strains did not grow on YPRAA plates (Figure 2B). These results confirmed that the signal peptide of Ps87 was functional, but the N-terminus of Mg87 was not. These results matched the SignalP3.0 predictions, but not predictions from two other popular prediction program iPSORT and PSORTII (Figure 2A).

### Ps87 can translocate into plant cells via a conserved RXLR-like motif

**Figure 3:**
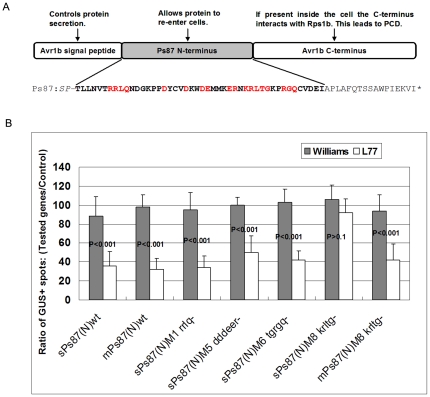
Cell re-entry assays of *Puccinia striiformis* Ps87 protein by particle bombardment mediated transient expression. (A) Structure of recombined Avr1b protein. Predicted Ps87 translocation domain (in bold) was fused to Avr1b by replacement of the Avr1b RXLR-dEER domain. Residues subjected to mutagenesis are labeled in red. (B) Cell re-entry activities of Ps87 fused Avr1b proteins carrying (s) or lacking (m) the secretory leader and/or with mutations in RRLQ (RRLQ to aaaa), DDDEER (DYCVDKWDEMMKER to aYCVaKWaaMMKaa), TG-RGQ (TGKPRGQ to aaKPaaa) and KRLTG (KRLTG to aaaaa) motifs. Effector re-entry activities were measured by double-barreled particle bombardment in which parallel bombardments with a beta-glucuronidase (GUS) reporter gene, with and without the *Avr1b* fusion were compared for the reduction of cell death in the presence of resistance gene *Rps1b* (cultivar L77-1863) or its absence (rps; cultivar Williams). Averages and standard deviations shown are from 14–16 pairs of bombardments. *P* values were calculated using the Wilcoxon rank sum test.

Two predicted RXLR-like translocation motifs, RRLQ and KRLTG, were identified based on functional RXLR variants validated in the *P. sojae* effector Avr1b [24], suggesting that Ps87 might be able to enter plant cells. To validate the prediction, the N-terminus of Ps87, encompassing the two motifs, was inserted between the secretory leader and the C-terminus of Avr1b, replacing the RXLR domain of Avr1b (Figure 3A). Transient expression of the *Ps87*-*Avr1b* fusion in soybean leaves carrying *Rps1b* triggered cell death, indicating that the N-terminus of Ps87 is capable of translocating the Avr1b C-terminal reporter back into soybean leaf cells (Figure 3B). The RRLQ and DDDEER motifs in Ps87 appeared quite similar to the RXLR-dEER bipartite protein translocation motif of oomycete effectors, but the alanine substitutions in these two motifs did not abolish the cell killing activity of the Avr1b reporter as measured by the reduction of beta-glucuronidase (GUS) positive spots. However, mutations in the motif KRLTG did lead to reduced Avr1b-mediated cell death in *Rps1b* leaves, suggesting that KRLTG was required for re-entry of the fusion protein. The interaction of the leaderless Ps87 fused Avr1b with Rps1b soybean was unaffected by mutations in the KRLTG motif, indicating that the KRLTG motif present in the Ps87 N-terminus was not involved in the interaction between Avr1b and Rps1b. KRLTG is well conserved in other fungal homologs, typically as RRLTG (Figure 1A). Another conserved motif, RGQ, did not appear to be involved in translocation.

### The conserved motif of *P. striiformis* Ps87 is sufficient to target GFP to soybean root cells

**Figure 4:**
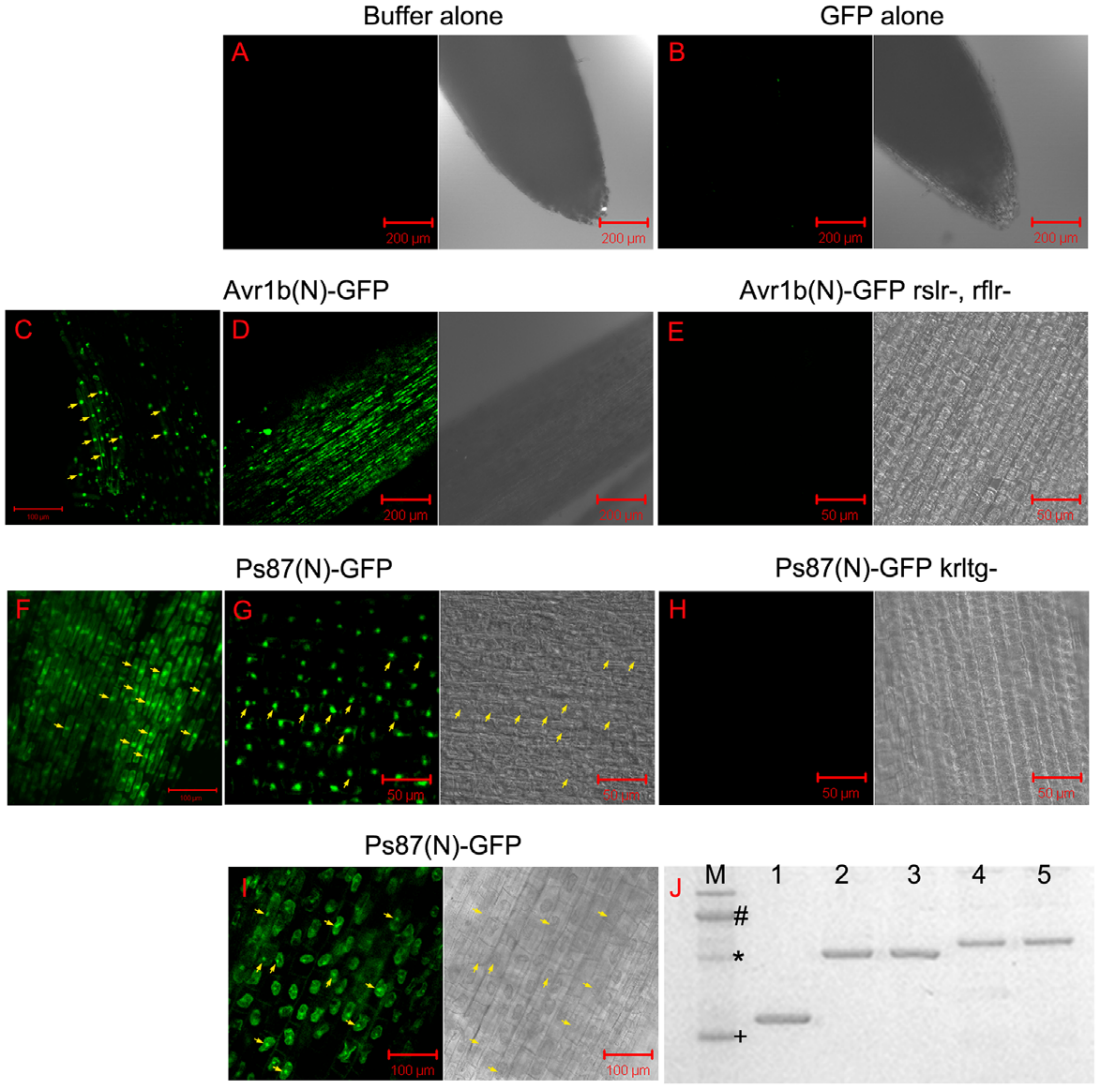
Root uptake assay of *Puccinia striiformis* protein Ps87 and confirmation of cell entry motif KRLTG. Entry of wild-type and mutant Ps87-GFP or Avr1b-GFP fusion proteins into soybean root cells. The paired light micrographs and fluorescence optical sections are from the same root tips in each case. All expressed GFP proteins fluoresce normally under UV illumination. Fluorescent micrographs were taken with the same photographic exposure in every case. Left panels, fluorescence images; right panels, light micrographs. (A–B) The buffer (A) and GFP (B) alone. Scale bars, 200 µm. (C) The Avr1b N-terminal uptake signal fused GFP. Scale bars, 100 µm. (D) The Avr1b N-terminal uptake signal fused GFP. Scale bars, 200 µm. (E) The Avr1b rslr^−^ and rflr^−^ nonfunctional mutant. Scale bars, 50 µm. (F) The Ps87 N-terminal uptake signal fused GFP. Scale bars, 100 µm. (G) The Ps87 N-terminal uptake signal fused GFP. Scale bars, 50 µm. (H) The Ps87 krltg^−^ nonfunctional mutant. Scale bars, 50 µm. (I) The Ps87 N-terminal uptake signal fused GFP. Photograph was taken 15 min after plasmolysis of the roots with 3 M NaCl. Scale bars, 100 µm. (J) Protein gel electrophoresis analysis of GFP fusion proteins partially purified from *E. coli* cells: lane 1, GFP alone; lane 2, GFP fused to the N-terminus of mature wild-type Avr1b protein; lane 3, same as lane 2 with RXLR motifs mutation; lane 4, GFP fused to the N-terminus of mature wild-type Ps87 protein; lane 5, same as lane 4 with KRLTG mutation; The left lane contained molecular mass markers (M). Size markers: # 50 kDa; * 40 kDa; +30 kDa.

A root uptake assay employing green fluorescent protein (GFP) fused to candidate effectors was previously employed to visualize specific translocation activity of the effectors [23], [24]. The predicted host-targeting signals of Ps87 and Avr1b were fused to GFP, expressed in *Escherichia coli* and partially purified (Figure 4J). As shown in Figure 4, both the RXLR domain of Avr1b and the N-terminus of Ps87 containing KRLTG carried the GFP fusion protein into soybean root tip cells in the absence of pathogens (Figure 4C, D, F, G). The GFP protein accumulated inside of several layers of the root tip cells, whereas buffer and GFP alone did not produce any fluorescence, confirming that uptake was specific (Figure 4A, B). The characteristic accumulation of GFP in the nuclei of the roots cells verifies that the GFP is located inside the cells. Furthermore, the GFP signal remained in the cytoplasm and nuclei rather than the intercellular spaces when the soybean root tissues were plasmolyzed with 3 M NaCl (Figure 4I). Mutations in the KRLTG motif of Ps87 as well as the RXLR motifs of Avr1b abolished accumulation of GFP fusion protein signals inside the soybean root cells (Figure 4E, H). Therefore the results of the GFP fusion protein uptake assay confirm the results from the bombardment experiments.

### Functional characterization of *P. striiformis* Ps87

**Figure 5:**
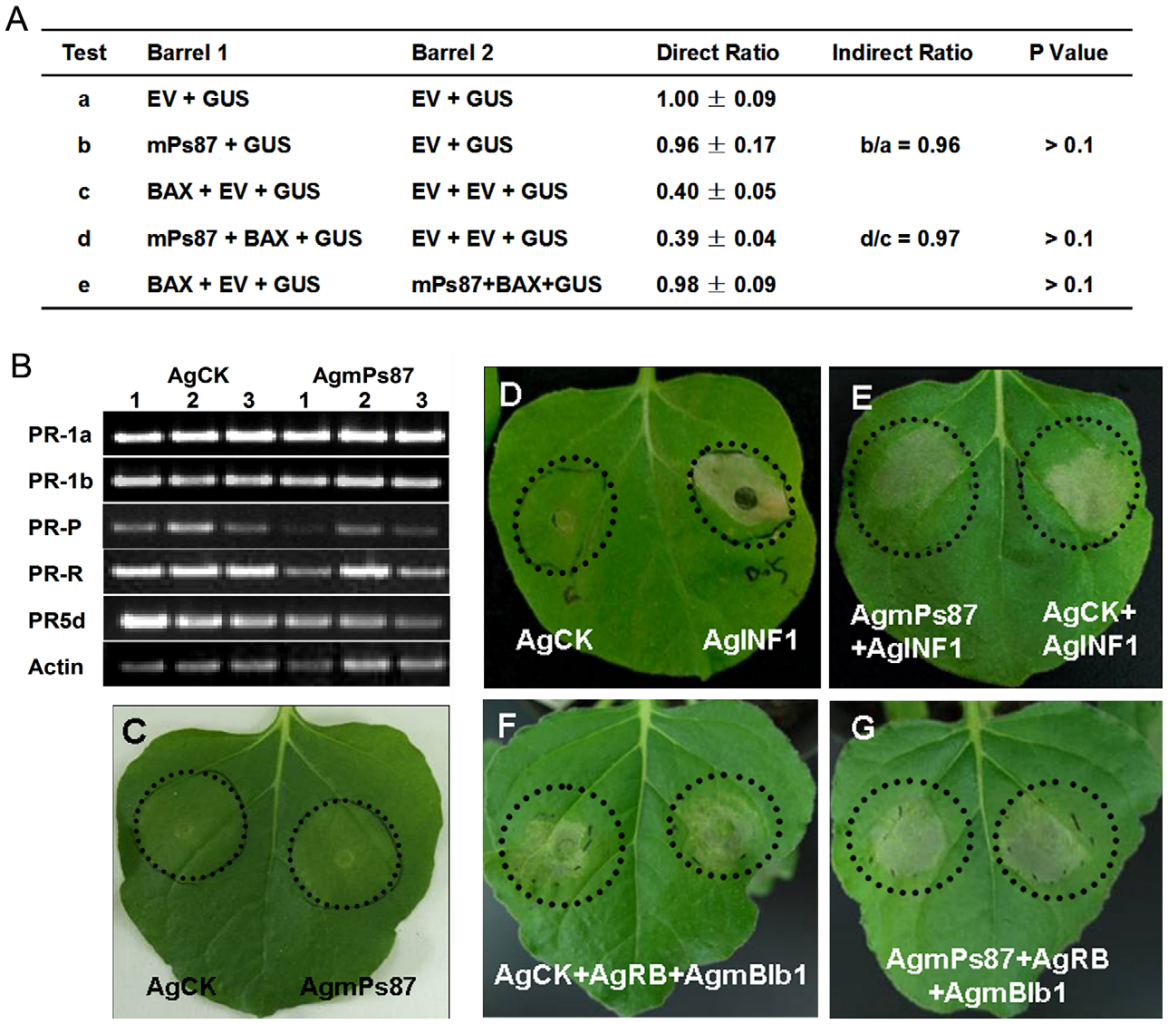
Transient expression of Ps87 does not alter defense responses. The *Puccinia striiformis* protein Ps87 neither induced defense responses nor suppressed PAMP (INF1), BAX or effector (Avrblb1) triggered defense responses in *Nicotiana benthamiana*. (A) Induction or suppression of cell death in soybean leaves. Mature Ps87 was tested for cell death induction and suppression of cell death using both the direct and indirect double-barrel bombardment assays as described [33]. Conclusions are based on statistical analysis of results from 14 to 16 leaves. The lower case letters indicates comparison of the two averaged ratios from the experiments. *P* values for the indirect comparisons were calculated from the log ratios using the Wilcoxon rank sum test. *P* value for the direct comparison (test e) was calculated from the log ratios using the Wilcoxon signed ranks test. EV, empty vector. (B) Ps87 does not perturb *PR* gene expression. RT-PCR analysis of expression of pathogenesis-related genes in *N. benthamiana* leaves transiently expressing Ps87. Total RNA was isolated 1 d, 2 d and 3 d after infiltration (dpi) with AgCK and AgmPs87, respectively and was analyzed for transcript levels of pathogenesis related genes *PR-1a*, *PR-1b*, *PR-P*, *PR-R* and *PR-5d* by RT-PCR. The *N. benthamiana* actin gene was used as a standard to normalize the results. (C) Ps87 does not trigger cell death. *N. benthamiana* leaves were infiltrated with *A. tumefaciens* cells carrying AgCK (empty vector) or AgmPs87. No obvious phenotypic changes were observed on the treated leaves five days after infiltration, including cell death, chlorosis, water-soaking or crinkling. (D)–(E) Ps87 does not suppress INF1-triggered cell death. AgCK (left) or AgINF1 (right) were infiltrated into *N. benthamiana* leaves alone (D), or AgPs87 (left) or AgCK (right) were infiltrated into *N. benthamiana* leaves 24 hr before infiltration with AgINF1. Dotted lines indicate the leaf infiltration zones. Photos were taken five days after the last infiltration. (F)–(G) Ps87 does not suppress effector-triggered cell death. A mixture of two *A. tumefaciens* strains carrying AgCK and AgRB respectively, or a mixture of two *A. tumefaciens* strains carrying AgmPs87 and AgRB respectively, were infiltrated into the leaves and incubated for 24 hr before the Agro-infiltration sites were further infiltrated with *A. tumefaciens* cells carrying the Avrblb1 effector gene. Dotted lines indicate the leaf infiltration zones. Photos were taken five days after the last infiltration.

To determine possible functions of mature Ps87 protein (mPs87), we tested whether it suppresses defense responses triggered by PAMPs and effectors. The suppression of PAMP triggered immunity was analyzed using the *P. infestans* INF1 elicitin [32] and suppression of cell death generally was analyzed using BAX, a pro-apoptotic protein from mouse that triggers an HR-like cell death response in plants [5], [33]. First, we infiltrated *A. tumefaciens* strains carrying plasmids AgmPs87 (encoding mature Ps87 protein) or AgCK (empty vector control) in *Nicotiana benthamiana*. After 24 hr, infiltration sites were challenged by infiltration of *A. tumefaciens* strains carrying plasmid AgINF1, and cell death symptoms were observed five days later. AgmPs87, like AgCK, never impaired INF1-induced HR in *N. benthamiana* (Figure 5D, E). Moreover, agroinfiltrition of AgmPs87 did not produce cell death in *N. benthamiana* leaves (Figure 5C). The ability of mature Ps87 protein to trigger cell death or to suppress BAX-induced cell death in soybean was evaluated by using the leaf bombardment assay (Figure 5A). The results showed that transient expression of mPs87 produced equal numbers of GUS positive spots compared with the empty vector, indicating that mPs87 was not a cell death inducer. Furthermore, the cell death inducing activities of BAX protein was not affected by mPs87 either, indicating that mPs87 was unable to suppress cell death triggered by BAX.

We further investigated whether Ps87 could suppress effector-triggered immunity by taking the advantage of cloned *P. infestans* effector gene *Avrblb1* [13] and the corresponding potato resistance gene *RB* [34]. *Agrobacterium*-mediated transient expression assays showed that Ps87 did not inhibit cell death triggered by recognition between transiently expressed Avrblb1 and RB proteins (Figure 5F, G).

**Figure 6:**
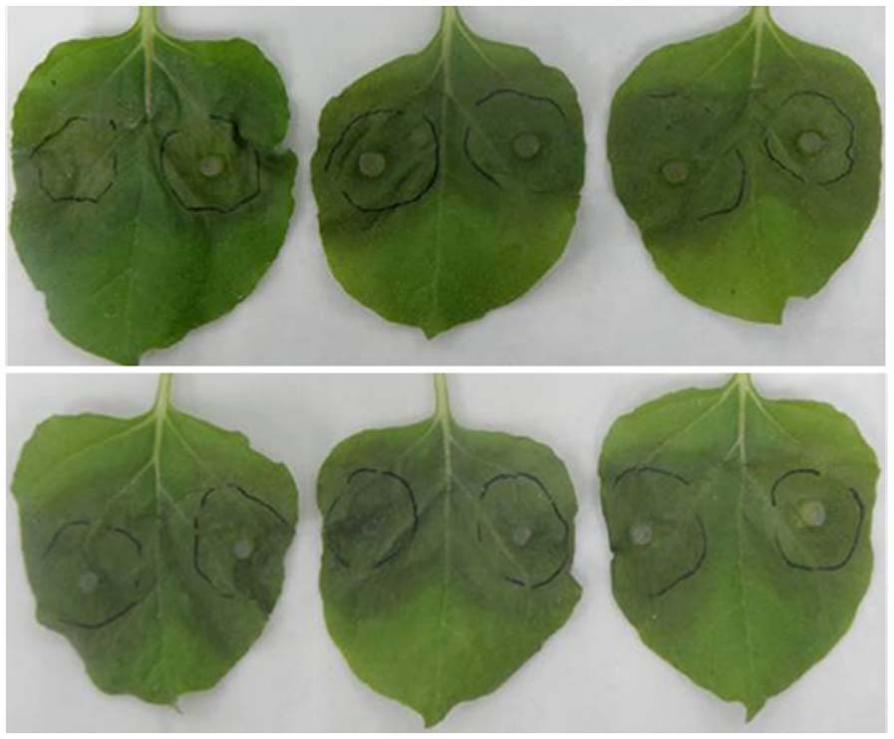
Transient expression of *Puccinia striiformis Ps87* does not affect infection of *Nicotiana benthamiana* by *Phytophthora parasitica*. Infiltration of *Agrobacterium tumefaciens* cells carrying AgmPs87 into *N. benthamiana* leaves was performed 24 hr before the infiltration sites were inoculated with *P. parasitica* mycelial plugs. Lesion development and pathogen colonization were observed and photographs were taken after four days. No significant differences in *P. parasitica* infection were observed between the infiltrated sites of AgCK upper panel and AgmPs87 lower panel; each panel shows three identical treatments.

To examine potential suppression activities of Ps87 on plant defense responses, we performed *Agrobacterium*-mediated transient expression of *Ps87* in tobacco leaves and quantified changes in the expression profiles of five defense associated genes, *PR-1a*, *PR-1b*, *PR-P*, *PR-R* and *PR-5d*. Most genes except *PR-R* (encoding protein with chitinase activity) displayed no substantial changes compared with that of empty vector at transcriptional levels following transient expression of *Ps87* at 1 d, 2 d and 3 d after infiltration (Figure 5B). We further tested whether the transient expression of *Ps87* affects plant susceptibility to the infection by *P. parasitica*. At 24 hr post-infiltration, the virulent *P. parasitica* strain Pp016 [28] was inoculated onto the infiltrated areas, then the water-soaked lesions were observed 24 hr later. These results showed that water soaked lesions developed equally on both empty vector control and the Ps87 treated leaves. Thus the infection process displayed no observable difference between Ps87 expressing leaves and control leaves (Figure 6).

## Discussion

Rust pathogens are one of the most important threats to the world's grain supply. The recent accumulation of EST and genome sequence data from rust fungi has opened the possibility of identifying the repertoire of proteins, including secreted effectors utilized by the pathogens during infection. Here we have characterized a potential effector, Ps87, from the wheat stripe rust fungus, which was identified from an EST screen. *Ps87* was predicted to encode a secreted protein by the signal peptide prediction program SignalP3.0. We validated this prediction using the yeast signal sequence trap method [11], a method also used to validate secretion of the AvrM and AvrP4 effectors of the flax rust pathogen [8]. Given that Ps87 is likely to be secreted, and given its up-regulated expression in germinated urediniospores [31], Ps87 appears a likely candidate to be an effector that plays a role in plant infection by *P. striiformis*.

The Ps87 protein sequence is highly similar to that of the haustoria-enriched secreted protein from flax rust, Hesp767 [8]. On the other hand, database searches led to the identification of many conserved Ps87 protein homologs in various fungal plant pathogens as well as non-pathogenic fungi. The aligned protein members share strong similarity in the central region of the protein, but not at the N-terminus. The variation in the N-terminal regions of the proteins is reflected in the fact that the ascomycete homologs of Ps87 are predicted not to be secreted, a prediction that we validated in the case of Mg87. This surprising difference between the rust clade of Ps87 homologs and the ascomycete clade of Ps87 homologs suggests that Ps87 may have been recruited as an effector from an intracellular protein, through mutations in its N-terminal signal peptide. The extensive sequence conservation of the central domain of Ps87-like proteins suggests that they may serve important functions in fungal physiology, although blast searches revealed no compelling hints regarding the function of the protein family. If the protein family serves an important function, then either rust fungus has evolved to no longer require intracellular Ps87/Hesp-767 proteins in this role, or else a sufficient fraction of the proteins may remain inside the hyphae to support the intracellular function; the somewhat weak SignalP3.0 scores for the rust Ps87 homologs might possibly reflect this putative dual role.

We speculate that the function served by the Ps87 homologs inside fungal cells may corrupt related functions inside plant cells, to the benefit of the pathogen, although the current limited assays we have used have not uncovered an obvious effect on plant immunity. It is also possible that being important in fungal physiology as suggested from its extensive sequence conservation in diverse fungal species, the newly recruited potential effector Ps87 has no significant effect on plant immunity as determined in the transient expression assay with tobacco plants. Furthermore, population genetic analysis of *Ps87* may provide clues on whether it plays a role in the interaction with corresponding wheat *R* gene(s) in a classical gene-for-gene manner. Wheat-stripe rust interaction follows gene-for-gene hypothesis in which recognition is required for initiating disease resistance. Pathogen effectors recognized by host R proteins are subjected to strong selection and thus quite diverse in sequence in general. Further investigating how this conserved secreted protein contributes to the pathogen's fitness is likely to provide novel insights into the pathogenesis of the rust pathogens.

Two lines of evidence support the hypothesis that Ps87 can enter plant cells via an RXLR-like motif, [K/R]RLTG, which forms part of the conserved central core of the Ps87 protein family. In one series of experiments we used particle bombardment assays to test the ability of Ps87 to carry Avr1b protein back into soybean leaf cells. In the second series of experiments, we showed that Ps87 could deliver GFP into soybean root cells. Both assays revealed that the motif KRLTG, which is conserved in the other family members as RRLTG, is required for the cell entry activity of Ps87. We note that these experiments were done in soybean, where these assays are relatively easy to carry out. Future validation using wheat leaves will be important to confirm this activity of Ps87.

The motif [K/R]RLTG matches the RXLR-like motif defined by Kale et al. [24] through mutagenesis of the Avr1b RXLR motif. In the Ps87 motif, the K and L residues are direct matches for the motif. Furthermore, Kale et al. [24] showed that both RLGT and RLTQ are functional in the context of Avr1b, suggesting that the highly conserved RLTG portion of the Ps87 motif could also carry the cell entry function. In addition to KRLTG, a second RXLR-like motif was in the Ps87 N-terminus. This motif, RRLQ-DDDEER motif, is more similar to the oomycete RXLR and dEER motif pair than KRLTG, but is less conserved among other Ps87 family members fungal proteins and mutations did not have any measurable effect on cell entry. Inactive RXLR and RXLR-like motifs were also found among several oomycete and fungal effectors, which suggests that sequences flanking RXLR motifs are involved in protein cell entry [23], [24], [27].

Additional potential translocation motifs were noted based on homology among putative fungal effectors that may enter cells. For example, the [Y/F/W]XC motif is conserved in many candidate effectors from powdery mildew and rust fungi [35], and was speculated to be involved in effector entry into plant cells. However, there is currently no experimental evidence to support their role in protein translocation. For example, Ps87 has no conserved [Y/F/W]XC motif, nor do the flax rust effectors, AvrL567, AvrM, AvrP123 and AvrP4. In AvrL567, the motif RFYR was shown to mediate translocation of that effector in two independent studies [24], [25]. In AvrM, a region containing three RXLR-like motifs, but no [Y/F/W]XC motifs was identified as the translocation domain [25].

The diverse RXLR variants of fungi effectors may imply different protein translocation machineries into plant cells. It is certainly likely that the translocation domains in fungi and oomycetes have evolved multiple times independently. Alternatively, the various effectors may employ a universal transport system despite their primary sequence variation. Kale et al. [24] presented evidence that RXLR domains of oomycete effectors and RXLR-like domains of fungal effectors mediate host cell entry by enabling binding to cell surface phosphatidylinositol-3-phosphate (PI-3-P). Plett et al. [27] provided similar evidence regarding the ectomycorrhizal effector MiSSP7. However, some difficulties have been reported in detecting PI-3-P-binding by fungal [36] and oomycete [37] effectors. It remains to be determined if the Ps87 family utilizes lipid-binding to enter plant cells. Since the [K/R]RLTG motif lies within a conserved domain found in non-secreted family members, if this conserved domain does enable entry into plant cells through binding to a particular ligand such as a lipid, then possibly the domain also mediates localization within the fungal cell via binding to the same ligand. It is of interest in this context that PI-3-P also mediates protein localization within eukaryotic cells.

## Supporting Information

Table S1 Oligonucleotide primers used in this study and Constructs used for bombardment, agroinfiltration and protein expression in this study.
(DOC)
