# Supplementary material for: Rust Secreted Protein Ps87 Is Conserved in Diverse Fungal Pathogens and Contains a RXLR-like Motif Sufficient for Translocation into Plant Cells
Source: PLoS One. 2011 Nov 4;6(11):e27217. doi: 10.1371/journal.pone.0027217 (PMC3208592; doi:10.1371/journal.pone.0027217)
Supplement: Table S1 — Oligonucleotide primers used in this study and Constructs used for bombardment, agroinfiltration and protein expression in this study. (DOC) [file pone.0027217.s001.doc]

**Supporting information**

**Table S1.** List of primers used in this study.

| Primers | Sequence 5’→3’ |
| --- | --- |
| PUCF | GGAGAGGACACGCTGAAATC |
| PUCR | GCTCAACACATGAGCGAAAC |
| mPs87FLF | CAGATcccgggATGACTCTGTTGAACGTGACT |
| Ps87R | GATCTggtaccTCAAATGACCTTTTCGATTG |
| Ps87NF | ATCGccatggGACCGGTGCCCTGGGCAAG |
| Ps87NR1 | CCACAGGTCAGTCACGCTGAAGGTTATCGGGTTG |
| Ps87NF1 | GTTAGCCGCTGCGGCAGTCACGTTCAACAG |
| Ps87M1F | GACTGCCGCAGCGGCTAACGATGGAAAGCCT |
| Ps87M5R | CATCATTGCAGCCCACTTGGCGACACAATAAGCTGGAGGCTTTCCATCGTTCTG |
| Ps87M5F | CCAAGTGGGCTGCAATGATGAAAGCGGCAAACAAACGATTGACTGGAAAAC |
| Ps87M6R | CATGCAGCGGCAGCGGCTGCAGCCAATCGTTTGTTTCTC |
| Ps87M6F | GGCTGCAGCCGCTGCCGCTGCATGTGTCGATGAAATAG |
| Ps87M8R | GTTTTGCAGCCGCTGCTGCGTTTCTCTCTTTCATCATTTC |
| Ps87M8F | GAAACGCAGCAGCGGCTGCAAAACCTCGAGGGCAATGTGTC |
| SUCPs87F | CTTAAgaattcATGCCGGTGCCCTGGGCAAG |
| SUCPs87R | GAACctcgagAGTCATTCCAGAGACTCCGAAC |
| SUC1bF | CTTAAgaattcATGCGTCTATCTTTTGTGC |
| SUC1bR | GAACctcgagAGTCATTGCGTTGCAGGTC |
| SUCMg87F | CTAAgaattcATGCCTGTTCCCTTCGAG |
| SUCMg87R | GAACctcgagGATACCGGCACCCGAAATG |
| AgINFF | CTAGcccgggATGGCCACCACGTGCACCAC |
| AgINFR | GATCTggtaccTCATAGCGACGCACACGTAG |
| AgmBlb1F | CTAGCcccgggATGGTTTCATCCAATCTCAAC |
| AgmBlb1R | GTGCAggtaccCTAGCTAGGGCCAACGTTTTTATC |
| pR9Ps87(N)wtF | GATCTagatctACTCTGTTGAACGTGACTCGGAG |
| pR9Ps87(N)wtF | CATATGGATAGCCGGACATGGTGGATCTGAATTCTATTTCATCGACACATTG |
| pR9Ps87(N)M8F | GATCTagatctACTCTGTTGAACGTGACTCGGAG |
| pR9Ps87(N)M8R | CATATGGATAGCCGGACATGGTGGATCTGAATTCTATTTCATCGACACATTG |
| GFPlinker | GGATCccatggAGCCAGCATAGTCTGGGACGTCATATGGATAGCCGGACATGGTG |

**Table S2.** Constructs used for bombardment, agroinfiltration and protein expression in this study.

| Construct | Vector | Construct stratagy |
| --- | --- | --- |
| sPs87(N)wt | PUC19 | The RxLR translocation region of secreted Avr1b was replaced by Ps87 N-terminus |
| mPs87(N)wt | PUC19 | The RxLR translocation region of mature Avr1b was replaced by Ps87 N-terminus |
| mPs87FLwt | PUC19 | Mature Ps87 was inserted into PUC19 vector with 35S promoter and polyA terminator |
| sPs87(N)1bM1 | PUC19 | The RxLR translocation region of secreted Avr1b was replaced by Ps87 N-terminus with alanine substitution of RRLQ motif |
| sPs87(N)1bM5 | PUC19 | The RxLR translocation region of secreted Avr1b was replaced by Ps87 N-terminus with alanine substitution of DDDEER motif |
| sPs87(N)1bM6 | PUC19 | The RxLR translocation region of secreted Avr1b was replaced by Ps87 N-terminus with alanine substitution of TGRGQ motif |
| sPs87(N)1bM8 | PUC19 | The RxLR translocation region of secreted Avr1b was replaced by Ps87 N-terminus with alanine substitution of KRLTG motif |
| mPs87(N)1bM8 | PUC19 | The RxLR translocation region of mature Avr1b was replaced by Ps87 N-terminus with alanine substitution of KRLTG motif |
| SUCPs87 | pSUC2 | Signal peptide fragments of Ps87 was fused in frame to the invertase gene in the pSUC2 |
| SUC1b | pSUC2 | Signal peptide fragments of Avr1b was fused in frame to the invertase gene in the pSUC2 |
| SUCMg87 | pSUC2 | First 25 Amino acids of Mg87 was fused in frame to the invertase gene in the pSUC2 |
| AgmPs87 | pCambia0380 | Mature Ps87 was inserted into pCambia0380 with 35s promoter and polyA terminator |
| AgINF1 | pCambia0380 | Phytophthora infestans INF1 was inserted into pCambia0380 with 35S promoter and polyA terminator |
| AgmBlb1 | pCambia0380 | Phytophthora infestans effector AvrBlb1(mature) was inserted into pCambia0380 with 35S promoter and polyA terminator |
| AgRB | pCambia0380 | Solanum bulbocastanum resistance gene RB was inserted into pCambia0380 with 35S promoter and polyA terminator |
| pR9-87(N) | pR9 | Ps87 N-terminus was fused to GFP in the protein expression vector pR9 |
| pR9-87(N)M8 | pR9 | Ps87 N-terminus with alanine substitution of KRLTG motif was fused to GFP in the protein expression vector pR9 |
| pR9-1b(N) | pR9 | Avr1b N-terminus was fused to GFP in the protein expression vector pR9 |
| pR9-1b(N)M3 | pR9 | Avr1b N-terminus with alanine substitution of RxLR-dEER motif was fused to GFP in the protein expression vector pR9 |
| pR9-GFP | pR9 | Enhanced GFP was inserted into protein expression vector pR9 |
